# Degeneration-Driven and Load-Modulated Fluid-Driven Viscoelasticity of the Human Intervertebral Disc: A Probabilistic Biphasic Swelling Modeling Study

**DOI:** 10.3390/bioengineering13030312

**Published:** 2026-03-09

**Authors:** Zhongwei Sun, Yixuan Dang, Changwen Mi, Jie Gu, Jiabao Pan

**Affiliations:** 1 School of Mechanical and Automotive Engineering, Anhui Polytechnic University, Wuhu 241000, China; zw-sun@ahpu.edu.cn; 2 Jiangsu Key Laboratory of Engineering Mechanics, School of Civil Engineering, Southeast University, Nanjing 210096, China

**Keywords:** lumbar intervertebral disc, fluid-driven viscoelasticity, degeneration, viscoelastic load levels, rheological model

## Abstract

Intervertebral disc (IVD) viscoelasticity is governed primarily by fluid transport driven by coupled osmotic and mechanical pressure gradients. Disc degeneration disrupts this balance through glycosaminoglycan loss and reduced cartilage endplate permeability. However, how degeneration interacts with compressive loading to regulate fluid-driven viscoelastic behavior at the whole-disc level remains unclear. To address this gap, a probabilistic biphasic swelling finite element framework was employed to simulate fluid-driven viscoelastic behavior of the IVD. Fifty discs were generated by varying anterior–posterior length, lateral width, nucleus pulposus volume ratio, wedge angle, and disc height. These discs were subjected to swelling, creep, and relaxation protocols under multiple compressive magnitudes for both healthy and degenerated conditions. Time-dependent responses were quantified using rheological models comprising two viscoelastic elements and one elastic element. Predicted intradiscal pressure, disc height, and viscoelastic responses fell within reported experimental ranges. Degeneration primarily governed fluid-dependent behavior. It reduced osmotic pressure, limited fluid mobility, and delayed axial equilibration. These changes decreased swelling displacement, increased creep deformation, and prolonged characteristic time constants, while minimally affecting instantaneous elastic response. In contrast, loading magnitude modulated the extent of viscoelastic deformation and progressively reduced degeneration-related differences in long-term creep displacement and long-term relaxation time constant. Collectively, degeneration governs fluid-dependent viscoelastic mechanisms, whereas loading magnitude modulates their expression. This study systematically examines how degeneration and load magnitude interact to regulate fluid-driven viscoelastic behavior of the IVD. By combining probabilistic geometry with biphasic swelling mechanics, it addresses a critical gap in understanding load–degeneration interactions in disc hydration-dependent mechanics.

## 1. Introduction

**Figure 1:**
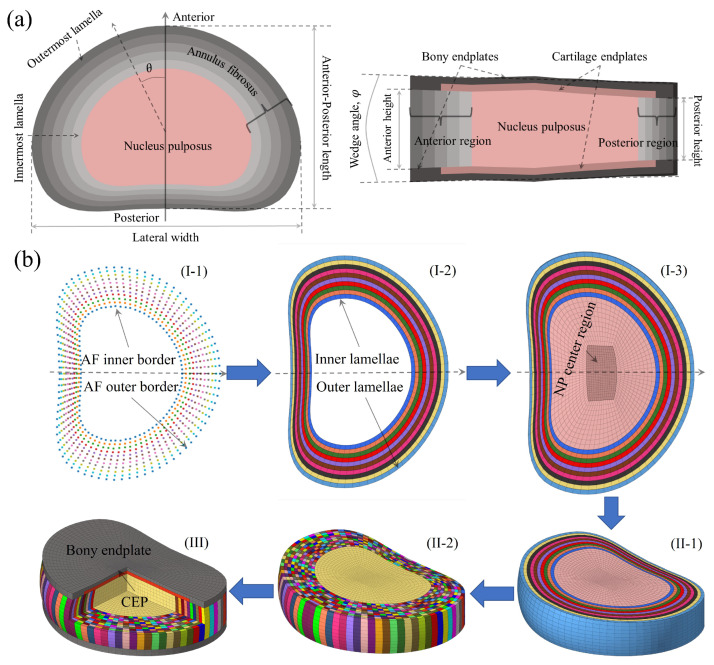
Illustration of the parameterization of disc morphology (**a**) and the workflow of the meshing process (**b**). The three stages identified in (**b**) are designated as I, II, and III, respectively. More detailed information about the parameterization and the meshing process, please refer to our previous research [6].

The intervertebral disc (IVD) is a hydrated fibrocartilaginous structure composed of a gelatinous nucleus pulposus (NP), a fiber-reinforced annulus fibrosus (AF), and superior and inferior cartilage endplates (CEPs) (Figure 1a). Disc height shows clear diurnal variation [1]. It decreases during daytime loading and recovers during nighttime unloading. This behavior reflects the viscoelastic nature of the IVD. Disc viscoelasticity arises from both the intrinsic viscoelastic behavior of the solid collagenous–proteoglycan matrix and fluid flow through the tissue [2]. The fluid-driven component is dominant and accounts for approximately ∼75% of the diurnal height change [2]. The present study, therefore, focuses on this fluid-dependent mechanism. Fluid-driven viscoelasticity is governed by mechanical loading and osmotic pressure. Therefore, variations in this behavior reflect both load magnitude and degeneration state. Although disc viscoelasticity has been widely studied [3,4,5], how degeneration alters fluid-driven responses, and how these changes are modulated by compressive load magnitude, remain incompletely understood. Importantly, prior studies have typically examined degeneration or load magnitude in isolation. A systematic evaluation of their interaction in governing fluid-driven viscoelastic mechanisms is still lacking.

Disc degeneration has been shown to accelerate creep and stress relaxation responses [7]. This acceleration is largely associated with reductions in glycosaminoglycans (GAGs) content [8]. Loss of GAGs reduces fixed charge density (FCD), further reduces osmotic pressure within the disc, and diminishes the pressure gradient with surrounding tissues, thereby suppressing fluid flow. Degeneration also alters CEP microstructure, including proteoglycan depletion and calcification, which reduce permeability and further restrict fluid transport [9,10]. Together, these changes decrease disc hydration and impair fluid-dependent viscoelastic function.

Numerous in vitro studies have examined disc viscoelasticity and NP mechanics [11,12,13]. A subset of studies has altered bath osmolality by adjusting salt concentration to manipulate the osmotic gradient [5,14]. These studies demonstrated that reductions in osmotic gradient substantially modify creep and recovery behavior. While this approach isolates osmotic effects, it does not fully represent degeneration, which also alters CEP permeability [10]. Degeneration therefore modifies disc viscoelasticity through coupled changes in osmotic pressure and transport resistance.

In addition to these in vitro approaches, O’Connell et al. [3] evaluated changes in rheological parameters across degeneration grades. Their dataset included only twelve lumbar discs. The discs were divided into five MRI-based grades, resulting in only two to three discs per grade. This small sample size limits robust characterization of degeneration-related trends. Moreover, many in vitro studies have examined viscoelastic behavior using animal discs [15,16,17]. Yet fluid-driven, time-dependent responses may be sensitive to geometry, structural organization, and biochemical composition. Thus, creep models obtained from animal discs cannot be directly extrapolated to human discs. This is especially true when drawing inferences about degeneration.

Numerical simulation can overcome limitations imposed by specimen availability and variability. This approach has recently been applied to complex biomechanical problems [18]. Investigation of degeneration-relevant viscoelasticity requires three elements. Constitutive models must capture biphasic swelling mechanics. A standard biphasic formulation lacks osmotic swelling driven by GAGs and, therefore, cannot represent proteoglycan-mediated hydration or its degeneration-induced alterations. Disc geometry must be parameterized to enable population-level simulations. Geometric variability can alter fluid pressure gradients by modifying transport path length, endplate surface area, and internal stress distribution, thereby influencing fluid redistribution. Degeneration must be represented in a physiologically meaningful manner.

To date, no numerical study has examined IVD fluid-dependent viscoelasticity while integrating all three elements within a unified framework. This gap limits mechanistic understanding of how degeneration and mechanical loading jointly regulate hydration-dependent time-dependent behavior. Our group has previously established the necessary foundations. We identified the ultrastructural collagen reinforcement of the AF, including oriented collagen fibers (OCFs) and nanoscale elastic fibers (NEFs) [19]. We developed biphasic swelling constitutive models incorporating ultrastructural reinforcement and solid–fluid interactions [20]. We further established parameterized disc geometry and an automated probabilistic simulation framework [6]. Finally, we proposed and validated a degeneration modeling strategy based on reduced FCD and decreased CEP permeability [21]. Together, these developments enable systematic investigation of degeneration-dependent fluid-driven viscoelasticity in the human lumbar disc.

The magnitude of compressive load applied to the IVD varies widely with posture, body weight, muscle activity, and external loading. However, most studies have used a single compressive load level. This restricts cross-study comparison and limits generalizability across load magnitudes [22,23]. As demonstrated by extant load-dependent studies, the relative contributions of the elastic component of creep behavior and recovery behavior components are modulated by compressive load magnitude [11,24]. These findings suggest that load level regulates fluid redistribution dynamics. However, how loading magnitude interacts with degeneration to regulate fluid-driven viscoelastic behavior remains unclear.

Therefore, this study has two aims. First, to determine how degeneration alters fluid-driven viscoelastic behaviors in the human lumbar IVDs. Second, to determine how compressive load magnitude modulates these degeneration-related changes.

## 2. Methods

### 2.1. Geometric Parameterization and Meshing Process

In our previous work, we described the parameterization and meshing of lumbar IVDs in detail [20]. Here, we summarize only the core principles, as shown in Figure 1b.

Five geometric features were parameterized. These included the anterior-posterior length (APL), lateral width, NP volume ratio, wedge angle, and disc height (Figure 1a). In the mid-axial plane, the NP boundary was defined using a specialized function [25]. The AF outer boundary was then obtained by adding AF thickness to the NP boundary. The AF was divided into twenty lamellae [26]. At the anterior and posterior extremes, lamellar thickness was set equal to the average lamellar thickness. The circumferential variation in lamellar thickness was assumed to be linear. Disc height was also assumed to vary linearly in both circumferential and radial directions.

In the present study, the element size choices were based on a rigorous convergence analysis previously conducted in our research [21]. The mean element size of 1.0 mm was designated for the innermost lamella, with the employment of 8-node trilinear hexahedral elements. In the axial direction, the element number of the AF was assigned to 10, while the element numbers of the CEPs and bony endplates (BEPs) were assigned to 3 and 2, respectively.

### 2.2. Sampling of Intervertebral Discs

As described above, we defined five geometric characteristics of the IVD: APL, lateral width, wedge angle, NP volume ratio, and disc height. In the preceding study [6], distributions for each parameter were sourced from the literature. In the present study, these distributions are summarised in Table 1. We then used the Latin hypercube sampling algorithm to generate the parameter dataset. This process yielded fifty geometric parameter sets. Each set was simulated under both healthy and degenerative conditions, producing two groups of finite element models. In total, one hundred lumbar IVDs were generated. All these models were simulated in FEBio [27].

### 2.3. Constitutive Models and Characteristics of Degeneration

Consistent with our previous work [20], we modeled intervertebral soft tissues (AF, NP, and CEPs) as biphasic swelling mixtures. The mechanical true stress was defined as the sum of solid stress, osmotic pressure, and fluid pressure (Equation (S1) in Supplementary Material). The solid stress originated from deformation of the solid matrix, and was quantified using the mixture strain energy density (Equation (S2)). In the AF, this strain energy density included contributions from the non-fiber matrix, OCFs, and NEFs (Equation (S3)). NEFs significantly affect AF tissue mechanics [19], but exert minimal influence at the whole-disc level [28]. Therefore, we considered only the dominant NEF fiber family with a principal direction of the OCF, as its content is the highest among NEF families (Table S1) [19]. In contrast, the NP solid matrix was assumed to contain only the non-fiber component, without NEFs. For the CEPs, the solid matrix included both the non-fiber matrix and NEFs.

The non-fiber matrix of all three soft tissues was modeled using the Holmes–Mow hyperelastic formulation (Equations (S4) and (S5)). A toe-linear stretch-only law (Equations (S5) and (S6)), defined in deformed configuration, was utilized to reproduce the non-linear behaviour of both the OCFs and NEFs. The elastic fibers in the CEPs were represented using an ellipsoidal fiber distribution model (Equations (S13)–(S15)). Fluid pressure was influenced by deformation-dependent permeability and external forces, and was defined using the Holmes–Mow permeability model (Equation (S12)). Osmotic pressure was governed by the tissue GAG content (Equations (S9) and (S10)), and was determined using the Donnan equilibrium model (Equation (S11)) with a constant saline concentration of the surrounding environment (0.15 M phosphate-buffered saline; 150 mmol/L). It is important to note that, in comparison with intervertebral soft tissues, the BEP demonstrates a considerably elevated initial permeability coefficient and elastic modulus. Consequently, the BEPs were modelled using a compressible neo-Hookean formulation with constant permeability (Equations (S7) and (S8)).

The constitutive models described above have been detailed in our earlier studies and in the Supplementary Material. The material properties of intervertebral soft tissues are listed in Table 2 and Table 3, and are based on previously validated experimental data. The FCD of the soft tissues and CEP permeability were reduced to represent degeneration, consistent with our prior framework [21]. In the present study, degeneration corresponds specifically to the most advanced grade (D3) defined in that work. Importantly, these grades are based on laboratory-observed compositional and structural alterations reported in the literature, rather than on clinical imaging classifications.

### 2.4. Loading Protocols

Three viscoelastic test protocols were applied to validate the finite element models and to quantify the effects of degeneration on the fluid-driven viscoelastic behaviors of IVDs. The protocols included swelling, creep, and stress-relaxation tests. To maintain tissue hydration and eliminate the effects of hydration time [29], all discs were assumed to be immersed in 0.15 M saline for a 30 h free-swelling period [21]. Before each creep test, a 20 N pre-compression was applied to the hydrated disc [3]. Creep tests were then performed under compressive loads of 500, 1000, and 1500 N. Three stress-relaxation tests were also conducted at axial engineering strains of 5%, 10%, and 15%. A 50 N pre-compression was applied prior to each relaxation test to reproduce the in vitro conditions [22,23]. Detailed loading descriptions are summarized in Table 4.

To apply compressive loading in a controlled manner, two rigid reference points were created near the superior and inferior surfaces of the IVD. They were defined as the “bc-dot” and the “loading-dot”, respectively, and were kinematically coupled to nodes of the inferior and superior BEPs. The “bc-dot” was fixed for all tests. In contrast, the “loading-dot” was allowed to move only along the IVD axis. To illustrate the fluid interface between the intervertebral disc and the external solution, a zero fluid pressure boundary condition was imposed on the external surface of the IVD for all protocols.

### 2.5. Data Analysis Methods

The free-swelling and creep tests were designed to characterize the time-dependent axial displacement (*u*). To quantify this response, we applied the double Voigt model:

(1) $$u=u_{e}+u_{1}\left(1-e^{-\frac{t}{\tau_{1}^{c}}}\right)+u_{2}\left(1-e^{-\frac{t}{\tau_{2}^{c}}}\right).$$

This equation enables the identification of parameters for the purely elastic ($u_{e}$), short-term ($\tau_{1}^{c}$ and $u_{1}$), and long-term ($\tau_{2}^{c}$ and $u_{2}$) responses.

For the relaxation tests, the time-dependent reaction force (*F*) was fitted using the double Maxwell model:

(2) $$F=F_{\infty }+F_{1}e^{-\frac{t}{\tau_{2}^{s}}}+F_{2}e^{-\frac{t}{\tau_{2}^{s}}}.$$

This model decomposes the force response into residual ($F_{\infty }$), short-term ($F_{1}$ and $\tau_{1}^{s}$), and long-term ($F_{2}$ and $\tau_{2}^{s}$) components.

Statistical analysis was performed using a custom Python script (Python 3.13, Python Software Foundation, USA). For each load level, we compared the elastic, residual, short-term, and long-term responses between healthy and degenerated groups using the Mann–Whitney U test. The same test was also used to assess differences among load levels. A significance threshold of 0.05 was applied. To address the issue of multiple comparisons, the Benjamin–Hochberg procedure was employed to control the false discovery rate. In this study, differences between healthy and degenerated groups are shown in the figures, whereas the *p*-values for comparisons across load levels are reported in the tables.

## 3. Results

### 3.1. Swelling Tests

**Figure 2:**
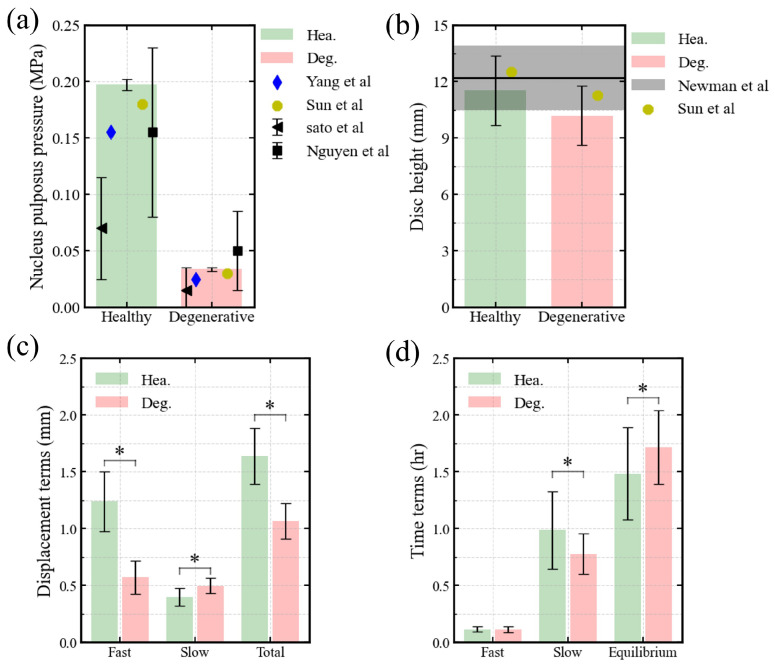
Simulation results of the swelling test protocol: Comparison of (**a**) intradiscal pressure and (**b**) hydrated disc height between the predictions of the present study and previously reported experimental [31,32], numerical data [21,30] and measured data from MRI [33]; and comparison of (**c**) displacement and (**d**) time components of the temporal evolution of axial displacement between healthy and degenerative disc groups. ‘*’ indicates p≤0.05.

Figure 2a,b demonstrates that our predictions agree well with both experimental results [31,32], computational results [21,30], and measured data from MRI [33], confirming the validity of our models. In both healthy and degenerated discs, the predicted intradiscal pressures fell within one standard deviation of experimental data (Figure 2a). The heights of healthy discs were within the ±95% confidence interval of literature values, while degenerated discs exhibited slightly reduced heights (Figure 2b). Figure 2c illustrates the distinct differences in axial displacement between the two groups. Figure 2d shows significant differences in the long-term and engineering equilibrium swelling time constants, whereas the short-term constants remained similar.

### 3.2. Creep Tests

**Figure 3:**
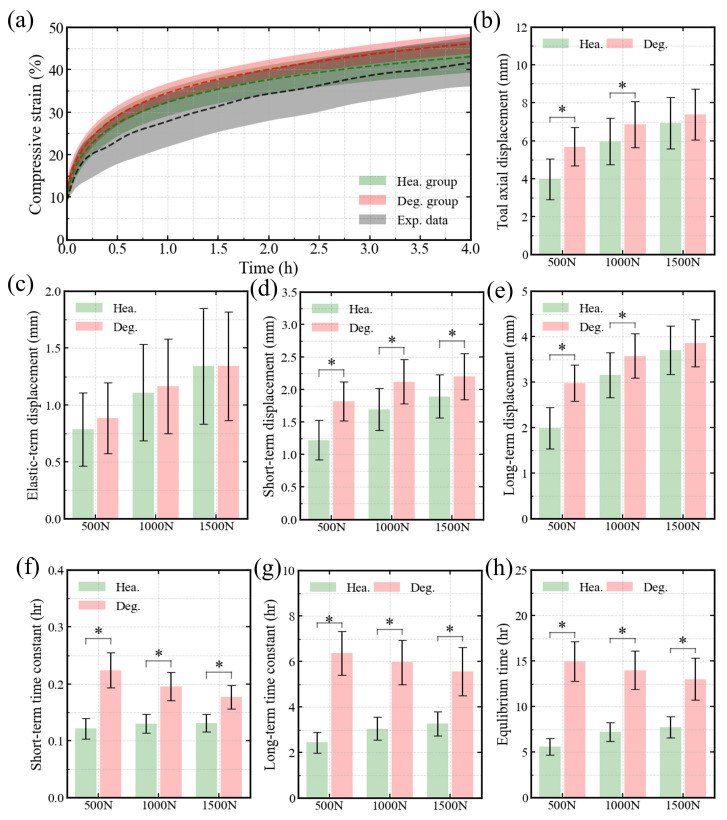
Simulation results of the creep test protocol: (**a**) Temporal evolution of axial compressive strain, with the ±95% confidence interval of experimental data [3]; comparison of (**b**) total axial displacement, (**c**) elastic-term displacement, (**d**) short-term displacement, and (**e**) long-term displacement in the temporal evolution of compressive displacement between healthy and degenerative disc groups; comparison of (**f**) short-term, (**g**) long-term, and (**h**) equilibrium time constants in the temporal evolution of axial displacement between healthy and degenerative disc groups. ‘*’ indicates p≤0.05.

Figure 3a illustrates the temporal evolution of axial compressive strain, showing that our predictions fall within the ±95% confidence interval of experimental results [3]. At all three load levels, Figure 3f–h reveals distinct differences between healthy and degenerated IVDs in the short-term, long-term, and equilibrium time constants. Regarding displacement, Figure 3d shows significant differences in short-term displacement across all load levels, while elastic deformation remained similar between groups (Figure 3c). The long-term and total displacements varied with load magnitude. At compression loads of 500 N and 1000 N, both long-term and total axial displacements differed markedly between healthy and degenerated discs, whereas no significant differences were observed at 1500 N (Figure 3b,e).

### 3.3. Relaxation Tests

**Figure 4:**
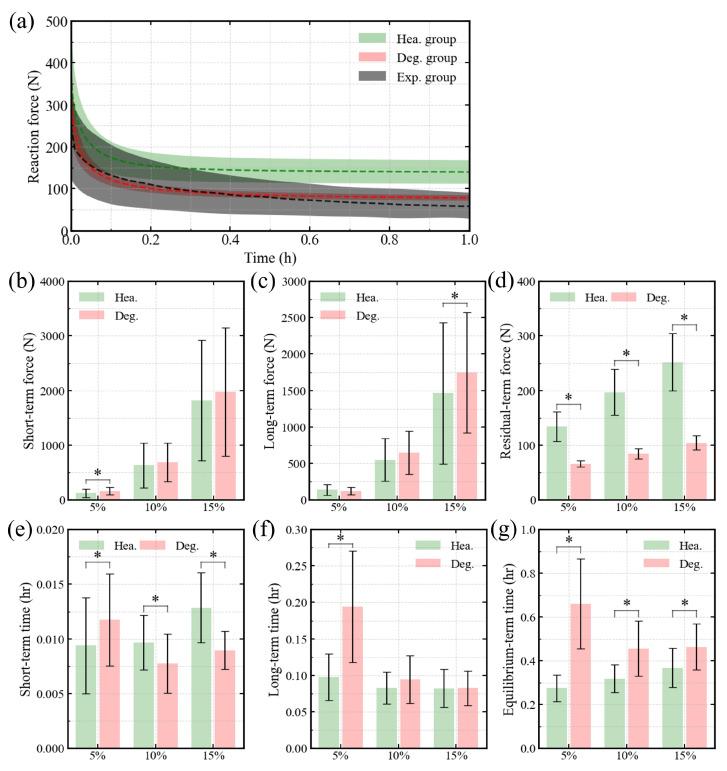
Simulation results of the relaxation test protocol: (**a**) Temporal evolution of reaction force, with the ±95% confidence interval of experimental data [22,23]; comparison of (**b**) short-term force, (**c**) long-term force, and (**d**) residual force in the temporal evolution of reaction force between healthy and degenerative disc groups; comparison of (**e**) short-term time constant, (**f**) long-term time constant, and (**g**) engineering equilibrium-term time constant in the temporal evolution of reaction force between the healthy and degenerative disc groups. ‘*’ indicates p≤0.05.

Figure 4a shows the time-dependent evolution of axial reaction force, indicating that predictions from the degenerated disc models fall within the ±95% confidence interval of experimental data [22,23]. Across all three compressive strain levels, Figure 4d,e,g demonstrates significant differences between healthy and degenerated discs in residual force, short-term time constants, and equilibrium time constants. For the relaxation test at 5% compressive strain, clear differences (p<0.05) were observed in both short-term force and long-term time constants (Figure 4b,f), whereas no significant differences appeared at higher strain levels. Regarding long-term reaction force, a significant difference was found only in the 15% strain test (Figure 4c).

### 3.4. *p*-Values Between Different Loading Levels

Table 5 summarizes the *p*-values of creep indicators across different compressive load levels. In the healthy group, both short- and long-term time constants showed no significant differences between the 1000 N and 1500 N loads (p=0.673 and p=0.054, respectively, Table 5). In the degenerated group, long-term time constants did not differ significantly between 500 N and 1000 N, or between 1000 N and 1500 N, but a significant difference was found between 500 N and 1500 N. The degenerated group also showed no significant variation in elastic, short-term, or equilibrium displacements between 1000 N and 1500 N.

Table 6 summarizes the *p*-values of relaxation indicators at different compressive strain levels. No significant differences were observed between 10% and 15% compressive strain in both healthy and degenerated groups for the long-term time constant. Within the degenerated group, equilibrium time showed no significant difference between 10% and 15% strain.

## 4. Discussion

This study examined how degeneration and loading magnitude jointly influence fluid-driven viscoelastic behavior in the human lumbar IVD. Using a probabilistic and automated simulation framework, we predicted the behaviors of healthy and degenerated discs and compared them with available experimental data. The predicted height of the degenerated disc lies near the lower bound of the reported experimental range (Figure 2b). This likely reflects that the modeled case represents a severely degenerated condition. Nevertheless, the overall predictions remain consistent with previous experimental and computational findings (Figure 2a,b, Figure 3a and Figure 4a). This supports the reliability and applicability of the adopted IVD models. By analyzing free swelling, creep, and stress relaxation within a unified biphasic swelling framework, we clarify how degeneration alters both deformation magnitude and time scale, and how these effects are modulated by mechanical loading. Across all simulated conditions, degeneration emerged as the dominant factor governing time-dependent behavior. In contrast, loading magnitude did not change the underlying mechanisms but regulated the extent to which degeneration-related effects were expressed. This distinction provides a consistent basis for interpreting the swelling, creep, and relaxation results. Importantly, this study integrates probabilistic geometry, degeneration modeling, and load variation within a single computational framework. This unified design allows degeneration- and load-dependent effects to be evaluated under consistent assumptions, thereby reducing methodological variability across conditions.

### 4.1. Selections of Rheological Model

Quantitative rheological models are commonly used to interpret the viscoelastic behavior of IVDs [13]. Previous studies have shown that disc deformation under loading typically consists of an instantaneous elastic response followed by time-dependent viscoelastic processes. The deformation rates of this viscoelastic process vary considerably between the short-term and long-term timescales [4,34]. This highlights the need to separate viscoelastic behavior into short- and long-term components. While various rheological formulations have been proposed, a key requirement for the present study was a model capable of consistently capturing these multiple viscoelastic phases, allowing systematic comparisons across degeneration states and loading conditions.

The five-parameter rheological model effectively describes disc recovery behavior [24]. By incorporating two viscoelastic units in addition to an elastic spring, this model enables separation of fast and slow viscous responses. Compared with simpler models, the five-parameter formulation provides a more complete representation of disc viscoelasticity without excessive complexity. This facilitates robust parameter identification and interpretation.

From a structural perspective, the use of two viscoelastic elements is also consistent with the composite nature of the disc. The NP and AF both exhibit time-dependent behavior, but their contributions to short-term and long-term deformation may differ. Within this phenomenological framework, the fitted parameters may reflect the combined influence of these components on overall disc behavior. Accordingly, double Voigt (Equation (1)) and double Maxwell (Equation (2)) representations were used to characterize creep and relaxation responses, respectively, allowing degeneration- and load-dependent changes in short- and long-term viscoelastic behavior to be evaluated in a consistent manner.

### 4.2. Effect of Degeneration

Direct experimental evidence on degeneration-dependent free swelling remains limited. Free swelling represents a simplified mechanical condition in which deformation is driven primarily by osmotic pressure, without external compression or stress redistribution. In contrast, creep and relaxation involve progressively more complex interactions among elastic deformation, fluid transport, and load-induced pressure gradients. Considering these conditions together provides a framework to isolate degeneration-related changes in fluid-dependent behavior and compare them across mechanical conditions.

Under free swelling, degeneration, modeled as reduced GAG content and decreased CEP permeability, resulted in reduced swelling displacement and prolonged equilibration (Figure 2b–d). Both the magnitude and the time scale of swelling were affected. These findings are consistent with prior studies reporting diminished deformation and delayed fluid-driven responses under reduced osmotic pressure [11,35]. In addition, these findings support recent work questioning the strict separation of displacement parameters as indicators of fluid volume and time constants as indicators of flow pathways [13,35]. Instead, the results suggest that degeneration modifies the coupled magnitude–time response of fluid transport, rather than selectively affecting a single aspect of swelling.

When external compression was introduced during creep, degeneration exerted minimal influence on the instantaneous elastic response (Figure 3c), consistent with prior experimental observations [12]. This indicates that elastic deformation is dominated by solid matrix compression and is relatively insensitive to changes in fluid pressurization caused by degeneration. In contrast, degeneration significantly increased time-dependent creep deformation (Figure 3b,d,e). As a result, total creep displacement increased, and creep stiffness decreased. This behavior can be attributed to reduced osmotic pressure and impaired fluid load sharing, which promote sustained solid matrix compression and delayed fluid redistribution [11,16]. The dominance of long-term creep deformation, accounting for more than half of the total displacement (Figure 3b,e), further highlights the central role of fluid-dependent mechanisms in degeneration-related creep behavior.

Degeneration also prolonged creep time constants across short-term, long-term, and equilibrium phases (Figure 3f–h). This indicates a slower approach to mechanical equilibrium. While some studies have reported increased NP permeability with degeneration, these effects appear to be offset at the whole-disc level by reduced osmotic driving forces and increased resistance to transport across the CEP [11,12]. Under the present modeling assumptions, endplate transport limitations and intervertebral soft tissues deformation-dependent permeability therefore dominated the temporal characteristics of creep, especially during long-term equilibration (Figure 3g).

Similar degeneration-dependent effects were observed during stress relaxation. Degeneration primarily reduced the disc’s ability to sustain equilibrium load (Figure 4d), while short- and long-term relaxation forces remained strongly load dependent (Figure 4b,c). This indicates that degeneration mainly compromises equilibrium internal pressurization rather than uniformly diminishing all viscoelastic force components [5,36]. Degeneration reduces osmotic pressure and water content, resulting in decreased disc height. At low compressive strain levels, osmotic pressure is comparable to the externally induced fluid pressure. Under these conditions, degeneration-related changes in osmotic pressure may significantly influence the short-term relaxation response, which may explain the difference observed at 5% strain (Figure 4b). At higher compressive strains, the degenerated disc becomes more compact, further reducing permeability and fluid mobility. This may explain the increased long-term reaction force observed at 15% strain for degenerated discs (Figure 4c). In parallel, degeneration significantly altered relaxation time scales. The equilibrium time constant was consistently prolonged (Figure 4g), reflecting delayed fluid–solid re-equilibration. Changes in short-term time constants were compressive strain dependent (Figure 4), suggesting a shift in the dominant relaxation mechanism with increasing compressive strain magnitude [5,37].

Overall, degeneration exerts a consistent influence across free swelling, creep, and relaxation by reducing osmotic driving forces and modifying fluid transport pathways. These changes have a limited impact on instantaneous elastic responses but strongly affect time-dependent deformation, force retention, and equilibration time scales. Together, these findings indicate that degeneration primarily alters the disc’s capacity to regulate fluid-dependent load sharing over time, rather than its instantaneous mechanical stiffness. The consistent trends observed across swelling, creep, and relaxation further support the internal coherence of the biphasic swelling formulation. By capturing osmotic pressure and transport resistance simultaneously, the model provides a mechanistic interpretation of degeneration-dependent time scales.

### 4.3. Effect of Loading Magnitudes

Loading magnitude markedly affected creep behavior by modulating the partitioning between elastic and time-dependent deformation. As compressive load increased, elastic deformation increased accordingly (Figure 3c), reflecting greater solid matrix compression [11]. Total creep deformation also increased, but in a nonlinear manner (Figure 3b). At higher loads, the rate of increase diminished, particularly in degenerated discs (p>0.05, Table 5). Under these conditions, creep displacements between healthy and degenerated discs became comparable (Figure 3b). This convergence suggests a progressive shift in load sharing from fluid pressurization toward solid matrix support as load magnitude increases. At elevated loads, osmotic and fluid pressures contribute less to long-term deformation, thereby attenuating degeneration-related differences in creep displacement [11,24] (Figure 3e).

Loading magnitude also influenced stress relaxation, but its effects were closely coupled with compressive strain level and degeneration state. At low strain levels, externally induced fluid pressures were insufficient to overcome intrinsic osmotic resistance, resulting in prolonged relaxation (Figure 4e–g and Table 6). At higher strains, fluid pressure exceeded osmotic resistance, accelerating early fluid outflow and shortening long-term relaxation (Figure 4f), particularly in degenerated discs. This effect is consistent with osmo-viscoelastic coupling reported in recent recovery and relaxation studies [5,16], where relaxation kinetics depend on both mechanical loading and osmotic conditions. In contrast to degeneration, loading magnitude did not consistently reduce residual force (Figure 4d). Instead, its primary influence was expressed through changes in relaxation time scales. Notably, loading magnitude did not uniformly amplify relaxation forces. Instead, its influence was primarily expressed through modulation of time constants, while degeneration consistently reduced residual force and prolonged equilibrium relaxation (Figure 4d,g). These results may indicate that loading magnitude shapes the pathway toward equilibrium, whereas degeneration determines the disc’s capacity to sustain long-term load support.

Taken together, these findings indicate that loading magnitude redistributes deformation mechanisms rather than uniformly scaling viscoelastic responses. Higher loads enhance elastic deformation and accelerate fluid-driven processes, while simultaneously reducing the sensitivity of creep and relaxation behavior to degeneration. In this context, degeneration governs the intrinsic fluid-dependent time scales, whereas loading magnitude determines how these processes are mechanically expressed under specific conditions. This mechanistic separation between intrinsic degeneration effects and load-dependent expression may facilitate future translation of computational findings to clinical loading scenarios, where both tissue condition and mechanical environment vary simultaneously.

### 4.4. Limitations

Several limitations should be acknowledged. First, all constitutive formulations were based on biphasic swelling mixture hyperelasticity. Under this assumption, time-dependent behavior was attributed exclusively to interstitial fluid transport, while intrinsic solid-phase viscosity was not explicitly modeled. This simplification may underestimate solid damping effects, particularly at short time scales. Future studies could address this limitation by incorporating viscoelastic constitutive laws for the solid matrix.

Second, the interpretation of rheological parameters relied on the commonly adopted assumption that disc viscoelasticity arises partly from fluid flow through hydrated tissues. Although this assumption is widely accepted, fluid pathways and exchanged fluid volumes were not directly quantified in the present study. As a result, the mechanistic interpretation of fitted parameters remains indirect.

Third, for both creep and relaxation simulations, the loading rate was kept constant. The potential influence of loading rate on the initial peak force and early fluid pressurization was not examined. Although this allowed degeneration and load magnitude to be evaluated independently, rate-dependent effects may also influence short-term mechanical response and should be considered in future studies.

Fourth, the analysis focused only on healthy and severely degenerated discs [21]. While this contrast allowed clear identification of degeneration-related effects, it does not capture the gradual evolution of viscoelastic behavior across intermediate degeneration stages. Including additional degeneration grades would provide a more complete description of this progression. Future work incorporating multiple degeneration grades would provide a more complete description of how disc viscoelasticity progressively changes with degeneration.

Finally, although geometries were probabilistically sampled to represent inter-individual variability, a formal uncertainty or sensitivity decomposition was not performed. The sampling strategy was intended to capture population-level variation rather than to enable structured variance partitioning among geometry, degeneration parameters, and loading magnitude. The relative contribution of each factor to overall response variance was therefore not explicitly quantified. Future work using variance-based or factorial sensitivity analyses would provide further quantitative insight.

## 5. Conclusions

Using a parametric and probabilistic biphasic swelling finite element framework, this study investigated how degeneration and loading magnitude jointly influence the fluid-driven viscoelastic behavior of human lumbar IVD. By examining free swelling, creep, and stress relaxation within a unified modeling approach, the results provide a coherent view of how degeneration modifies both the magnitude and the time scale of time-dependent mechanical responses. Across all loading scenarios, degeneration primarily affected fluid-dependent behavior rather than instantaneous elastic deformation. Degenerated discs exhibited reduced swelling capacity, increased creep deformation, and prolonged characteristic time constants, reflecting impaired fluid pressurization and delayed fluid redistribution. These changes are consistent with degeneration-induced reductions in osmotic pressure and endplate permeability, which limit the disc’s ability to sustain and restore internal fluid pressure. In contrast, loading magnitude acted mainly as a modulating factor. Increasing compressive load or strain enhanced viscoelastic deformation and progressively reduced differences between healthy and degenerated discs in long-term creep displacement and relaxation force. At higher loads, load sharing shifted toward the solid matrix.

Overall, the findings indicate that degeneration governs the intrinsic fluid-dependent viscoelastic mechanisms of the disc, whereas loading magnitude determines how strongly these mechanisms are expressed under specific mechanical conditions. This interaction offers a mechanistic explanation for the load-level dependence reported in experimental studies and underscores the need to consider degeneration severity and loading context jointly when interpreting disc viscoelastic behavior. It should be noted that the present findings primarily apply to time-dependent and low-frequency mechanical conditions characterized by fluid redistribution and osmotic equilibration. Degeneration-dependent behavior under high-frequency dynamic loading may involve additional mechanisms and warrants further investigation. It also should acknowledge that these findings may provide mechanistic insight into load-dependent degeneration processes.

## Figures and Tables

**Table 1:** The mean and standard deviation of the geometric parameters. It was hypothesised that each parameter would follow a Gaussian probability density function.

Parameters	Disc Height, mm	Anterior-Posterior Length, mm	Lateral Width, mm	Nucleus Pulposus Volume Ratio, %	Wedge Angle, °
Mean values	10.21	37.5	55.5	39.3	7.82
Standard deviation	1.8	6.21	6.3	5.5	6.07

**Table 2:** Material properties and permeability for bony endplates (BEPs), cartilage endplates (CEPs), nucleus pulposus (NP), and annulus fibrosus (AF). The parenthetical notation “H” and “D” denote healthy and degenerative models, respectively. Parameters identified with superscript “*a*” portray linear variation from the inner to outer (entries within parentheses) lamellae of the AF. $E_{m}$, ν, and $\beta_{m}$ are the parameters of the Neo–Hookean formulation for the BEPs or the Holmes–Mow model for soft tissues. $\beta_{m}$, *M*, and $k_{0}$ are the permeability parameters of soft tissues. $\varphi_{0}^{w}$ and $c_{f0}$ are the water volume fraction and the FCD of soft tissues in the initial configuration, respectively. The physical significance of all parameters was outlined in the Supplementary Information (see Equations (S1)–(S15)). The identification of these parameters’ values was achieved through the utilization of experimental data reported in previous literature [20,21].

Property	BEPs	CEPs (H)	CEPs (D)	NP (H)	NP (D)	AF (H)	AF (D)
$E_{m}$ (MPa)	12,000	0.25	0.328	0.065	0.065	0.027	0.027
ν	0.3	0.16	0.25	0.24	0.24	0.16	0.16
$\beta_{m}$	N.A.	1	1.06	0.95	0.95	0.09	0.09
*M*	N.A.	4.9	3.9	1.9	1.9	4.8	4.8
$k_{0}$ ($10^{-4}$ mm^4^/Ns)	$5\times 10^{4}$	5.56	2.5	5.5	5.5	16 (47) ^*a*^	16 (47) ^*a*^
$\varphi_{0}^{w}$	0.6	0.6	0.6	0.8	0.8	0.8 (0.7) ^*a*^	0.8 (0.7) ^*a*^
$c_{f0}$ (mmol/L)	N.A.	−326	−365	−300	−100	−300 (−100) ^*a*^	−150 (−100) ^*a*^
Φ	N.A.	1	1	1	1	1	1

**Table 3:** Intrinsic parameters of collagen fibers for different locations. Parameters for collagen fibers in the middle lamellae vary linearly from the inner to outer boundaries of the annulus fibrosus, remaining constant during the process of degeneration. Abbreviations: anterior outermost region (AO), anterior innermost region (AI), posterior outermost region (PO), posterior innermost region (PI). $s_{f}^{II}$ is the scale factor of the elastic fiber family, and is a measure of the elastic fibre content. The parameter of elastic fibers presented here is for the elastic fiber family with $s_{f}^{II}=1$ (Table S1). The moduli of the simplified elastic fiber model of the cartilage endplate, denoted $E_{f}^{ap}$, $E_{f}^{l}$, and $E_{f}^{a}$, respectively, represent the modulus in the anterior-posterior, lateral, and axial directions. The identification of these parameters’ values was achieved through the utilisation of experimental data reported in previous literature [19].

Property	Collagen Fibers				Elastic Fibers ($s_{f}^{\mathrm{II}}=1$)	Cartilage Endplate ($E_{f}^{\mathrm{ap}}=E_{f}^{l}=10E_{f}^{a}$)
	**AO**	**AI**	**PO**	**PI**		
Modulus, $E_{f}\left(E_{f}^{ap}\right)$, MPa	123.98	52.85	98.7	20.17	0.62	7.01
Rate of fiber stiffening, $\beta_{f}$	4.3	3.92	3.82	6.03	2.68	2.88
Critical stretch square, $I_{0}$	1.04	1.16	1.21	1.39	1.93	\

**Table 4:** Detailed specifications of the four test protocols conducted in the current work for the investigation of hydration-dependent responses of healthy and three degenerative disc models.

Test	Preload	Variable	Protocol	Comparative Experiment
Swelling	Non	Fixed charge density	Load from 0 to full in 1 s and then hold constant for 30 h	[30]
Pre-compression 20 N	Swelling	Compressive force	Load from 0 to 20 N under the rate of 1 N/s then hold constant for 30 h	[3]
Pre-compression 50 N	Swelling	Compressive force	Load from 0 to 50 N under the rate of 1 N/s then hold constant for 30 h	[22,23]
Creep	Pre-compression 20 N	Compressive force	Load from 20 N to 500, 1000, or 1500 N in 1 s, 2 s, or 3 s, respectively, and then hold constant for 30 h	[3]
Stress-relaxation	Pre-compression 50 N	Compressive strain	Load from 0% to 5%, 10% and 15% in 5 s, 10 s and 15 s, respectively, and then hold for 30 h.	[22,23]

**Table 5:** *p*-values of intervertebral disc creep responses under different compression loads.

Group	Indicators	500 N vs. 1000 N	500 N vs. 1500 N	1000 N vs. 1500 N
Healthy	Elastic-term displacement	0.000	0.000	0.023
	Shot-term displacement	0.000	0.000	0.004
	Long-term displacement	0.000	0.000	0.000
	Total displacement	0.000	0.000	0.001
	Short-term time	0.021	0.008	0.673
	Long-term time	0.000	0.000	0.054
	Equilibrium time	0.000	0.000	0.027
Degenerative	Elastic-term displacement	0.002	0.000	0.060
	Shot-term displacement	0.000	0.000	0.322
	Long-term displacement	0.000	0.000	0.020
	Total displacement	0.000	0.000	0.060
	Short-term time	0.000	0.000	0.001
	Long-term time	0.060	0.000	0.074
	Equilibrium time	0.027	0.000	0.038

**Table 6:** *p*-values of lumbar intervertebral disc relaxation responses under different compressive strain.

Group	Biomechanical Indicator	5% vs. 10%	5% vs. 15%	10% vs. 15%
Healthy	Residual force	0.000	0.000	0.000
	Short-term force	0.000	0.000	0.000
	Long-term force	0.000	0.000	0.000
	Short-term time	0.214	0.000	0.000
	Long-term time	0.023	0.022	0.923
	Equilibrium time	0.001	0.000	0.003
Degenerative	Residual force	0.000	0.000	0.000
	Short-term force	0.000	0.000	0.000
	Long-term force	0.000	0.000	0.000
	Short-term time	0.000	0.000	0.007
	Long-term time	0.000	0.000	0.086
	Equilibrium time	0.000	0.000	0.482

## Data Availability

The original contributions presented in this study are included in the article/Supplementary Material. Further inquiries can be directed to the corresponding author(s).

## Supplementary Materials

The following supporting information can be downloaded at: https://www.mdpi.com/article/10.3390/bioengineering13030312/s1, Table S1: The scaling factor $s_{f}$ and the concentration factor *b* for each elastic fiber’s family. Parameter values for the 45° and 135° fiber families were averaged. The spatial distributions of the scaling factor were obtained by linear interpolation along the radial direction for both the lamella and inter-lamellae layers. Abbreviations: AO (Anterior outer); AI (Anterior inner); PO (Posterolateral outer); PI (Posterior inner); LM (Lamella); ILM (Inter-lamellae).
