# Supplementary material for: Degeneration-Driven and Load-Modulated Fluid-Driven Viscoelasticity of the Human Intervertebral Disc: A Probabilistic Biphasic Swelling Modeling Study
Source: Bioengineering (Basel). 2026 Mar 9;13(3):312. doi: 10.3390/bioengineering13030312 (PMC13024115; doi:10.3390/bioengineering13030312)
Supplement: Supplementary file 1 [file bioengineering-13-00312-s001.zip › bioengineering-4178310-supplementary.pdf]

Degeneration-Driven and Load-Modulated Fluid- Driven Viscoelasticity of the Human Intervertebral Disc: A Probabilistic Biphasic–Swelling Modeling Study

Zhongwei Sun[a], Yixuan Dang[b], Changwen Mi[b], Jie Gu[a,*], Jiabao Pan[a,*]

[a] *School of Mechanical and Automotive Engineering, Anhui Polytechnic University, Wuhu, Anhui, 241000, China*

[b] *Jiangsu Key Laboratory of Engineering Mechanics, School of Civil Engineering, Southeast University, 2 Sipailou Street, Nanjing, 210096, Jiangsu, China*

S1 Constitutive models of solid components

The intervertebral disc (IVD) comprises several components, including the bony endplates (BEPs), cartilage endplates (CEPs), annulus fibrosus (AF), and nucleus pulposus (NP). To describe the mechanical behavior of these components, we employed a biphasic swelling theory framework. Within this framework, each tissue is conceptualized as a composite, consisting of a charged solid porous phase saturated by an interstitial fluid phase. The total Cauchy stress ($\boldsymbol{\sigma}$) is defined as follows:

$$\boldsymbol{\sigma} = -(p_f + p_{os})\boldsymbol{I} + \boldsymbol{\sigma}_s, \tag{S1}$$

where p_f is the fluid pressure, p_{os} the osmotic pressure and $\boldsymbol{\sigma}_s$ the solid stress. The solid stress originated from deformation of the solid matrix, and was quantified using the mixture strain energy density. This mixed law enables us to relate the overall mechanical response of a biomechanical structure to the behavior of its individual constituents, including collagenous fibers and the nonfibrillar matrix (NFM),

$$W_s = W_m + W_f, \tag{S2}$$

where W_s is the strain energy density of the mixed solid part, W_m and W_f are represent the contributions of nonfibrillar matrix and reinforcing fibers, respectively.

For the AF, the strain energy function of its solid component can be dissected into three distinct facets, including the contributions of the nonfibrillar matrix, the collagen fibers (W_f^I), and the elastic fibers ($W_f^{II-\psi}$):

$$W_s = W_m + W_f^I + \sum_{\psi} s_f^{II-\psi} W_f^{II-\psi} \tag{S3}$$

where ψ denotes the principal direction ($\boldsymbol{b}_0^{\psi}$) of an elastic fiber family and $s_f^{II-\psi}$ is the scaling factor that reflects the total number of fibers in the family.

For all intervertebral soft tissues, their nonfibrillar matrix was characterized using the Holmes-Mow hyperelastic constitutive model. Its strain energy density is expressed as:

$$W_m(I_1, I_2, J) = \frac{\lambda + 2\mu}{4\beta_m}\left(e^{\frac{\beta_m}{\lambda+2\mu}[-(\lambda-2\mu)(I_1-3)+\lambda(I_2-3)-(\lambda+2\mu)\ln(J^2)]} - 1\right), \tag{S4}$$

where β_m denotes the rate of exponential stiffening. The various parameters were listed in Table 2. The nonlinear behavior of the collagen fibers and nanosized elastic fibers in the annulus fibrosus was represented using a toe-linear stretch-only law,

$$W_f = \begin{cases} 0, & I_n < 1, \\ \frac{\xi_f}{\beta_f}(I_n - 1)^{\beta_f}, & 1 \leq I_n \leq I_0, \\ B_f(I_n - I_0) - E_f\left(\sqrt{I_n} - \sqrt{I_0}\right) + \frac{\xi_f}{\beta_f}(I_0 - 1)^{\beta_f}, & I_0 \leq I_n, \end{cases} \tag{S5}$$

where I_0 stands for the critical square of the fiber stretch (λ_0), β_f the toe-region power-law coefficient, I_n the square of fiber stretch in the deformed configuration. Such as, for a collagen fiber, $I_n = \boldsymbol{a} \cdot \boldsymbol{C} \cdot \boldsymbol{a}$, where $\boldsymbol{C}$ is the right Cauchy-Green deformation tensor, $\boldsymbol{a}$ the unit vector the collagen fiber in the deformed configuration. ξ_f and B_f are both functions of the linear-region elastic modulus (E_f), I_0, and the toe-region power-law coefficient (β_f),

$$\xi_f = \frac{E_f}{4(\beta_f - 1)}(I_0)^{-\frac{3}{2}}(I_0 - 1)^{2-\beta_f}, \qquad B_f = \xi_f(I_0 - 1)^{\beta_f - 1} + \frac{E_f}{2}(I_n)^{-\frac{1}{2}}. \tag{S6}$$

Specifically, for the BEPs, we utilized the compressible neo-Hookean model to simulate their constitutive behavior. This model is characterized by a hyperelastic strain-energy density, which can be expressed as:

$$W = \frac{\mu}{2}(I_1 - 3) - \mu \ln J + \frac{\lambda}{2}(\ln J)^2, \tag{S7}$$

where J stands for the determinant of the deformation gradient tensor ($\boldsymbol{F}$), I_1 the first invariant of the right Cauchy–Green deformation tensor ($C = \boldsymbol{F}^T\boldsymbol{F}$), λ and μ Lamé constants that relate to Young's modulus (E) and Poisson's ratio (v):

$$\lambda = \frac{Ev}{(1+v)(1-2v)}, \qquad \mu = \frac{E}{2(1+v)}. \tag{S8}$$

In this work, the BEPs were modeled as a biphasic neo-Hookean material with the Young's modulus $E = 12$ GPa and Poisson's ratio $v = 0.3$. In addition, a constant permeability of $k_0 = 5\ \mathrm{mm}^4/\mathrm{Ns}$ was chosen, in order to allow fluids to flow through.

S2 Donnan equilibrium model

In the biphasic-swelling theory, the glycosaminoglycan (GAG) content is related to the fixed charge density. Under the reference configuration prior to deformation, the fixed charge density is given by:

$$c_{f0} = \frac{c_g Z_c}{M_c}, \tag{S9}$$

where c_g is the GAG content, with the dimension mg/ml, and M_c and Z_c are the molecular

weight and number of charges of chondroitin-6-sulfate disaccharide. The latter two parameters were chosen as $M_c = 502.5$ gram and $Z_c = 2$ charges per repeating unit. Moreover, to reflect the loss of GAG content in degenerated IVD models, lower levels of initial fixed charge densities were assigned for the CEPs, AF, and NP (Table 1 of the manuscript).

Under the application of external loads, the instantaneous fixed charge density (c_f) evolves as a function of the deformation Jacobian (J), initial water volume fraction (φ_0^w) and initial fixed charge density (c_{f0}):

$$c_f = \frac{c_{f0}\varphi_0^w}{J - 1 + \varphi_0^w}. \tag{S10}$$

During the deformation process, the evolution of fixed charge density results in the osmotic pressure:

$$p_{os} = RT\Phi\left(\sqrt{c_f^2 + c_b^2} - c_b\right), \tag{S11}$$

where R is the gas constant, T the temperature, Φ the osmotic coefficient, and c_b the osmolarity of the external bath. During simulations, all tissues and the entire IVD were assumed to be immersed inside 0.15 M phosphate-buffered saline solution. For the ideal bath condition assumed in the current work, $\Phi = 1$ (Table 1 of the manuscript).

S3 Holmes-Mow permeability model

For the solid matrix of CEPs, AF and NP, an isotropic and deformation-dependent permeability behavior is also accommodated in terms of the Holmes-Mow permeability model (Holmes and Mow, 1990):

$$\boldsymbol{k} = k_0\left(\frac{J + \varphi_0^w - 1}{\varphi_0^w}\right)^2 e^{\frac{M}{2}(J^2-1)}\boldsymbol{I}, \tag{S12}$$

where $\boldsymbol{k}$ is the instantaneous hydraulic permeability tensor, k_0 and φ_0^w the initial isotropic hydraulic permeability and the water volume fraction in the reference configuration, and M the exponential coefficient of permeability. Both M and k_0 were determined by fitting to confined compressive experimental data (Cortes et al., 2014; Cortes and Elliott, 2012). They are tabulated in Table 1 of the main manuscript. It is noted that, for the CEPs, M and k_0 are different and in general decrease with the degeneration grade of the IVD (DeLucca et al., 2016). They are also tabulated in Table 1.

Under a quasi-static condition, the hydraulic permeability ($\boldsymbol{k}$) relates the volumetric flux of the fluid relative to the NFM, w, to the interstitial fluid pressure gradient, ∇p_f, according to the Darcy's law ($w = -\boldsymbol{k} \cdot \nabla p_f$). In general, the hydraulic permeability ($\boldsymbol{k}$) is a function of the deformation, as indicated by Eq. (S7).

S4 Ellipsoidal-fiber distribution model

The CEPs are fiber-reenforced composites. The fibers orient primarily parallel to the planes of the vertebrae. In addition, there are no significant differences in the lateral and anterior-posterior directions (DeLucca et al., 2016). In current work, the reenforcing fiber phase was modeled using an ellipsoidal fibers distribution model. The strain-energy density of this model is defined as:

$$\Psi_f(\boldsymbol{n}, I_n) = \xi(\boldsymbol{n})(I_n - 1)^{\beta(\boldsymbol{n})}, \tag{S13}$$

where I_n represents the instantaneous square of the fiber stretch, i.e., $I_n = \boldsymbol{N} \cdot \boldsymbol{C} \cdot \boldsymbol{N}$, with $\boldsymbol{N}$ being the unit vector along the fiber axis in the reference configuration and $\boldsymbol{n}$ the unit vector of fiber in the current reference, i.e., $\boldsymbol{n} = \boldsymbol{F} \cdot \boldsymbol{N}/\lambda_n$. In spherical coordinate system, the unit vector $(\boldsymbol{n})$ is measured by the longitudinal (φ) and latitudinal (θ) coordinates. With their help, the material parameters $\beta(\boldsymbol{n})$ and $\lambda(\boldsymbol{n})$ can be defined as:

$$\xi(\boldsymbol{n}) = \left(\frac{\cos^2\theta \sin^2\varphi}{\xi_1^2} + \frac{\sin^2\theta \sin^2\varphi}{\xi_2^2} + \frac{\cos^2\varphi}{\xi_3^2}\right)^{-\frac{1}{2}}, \tag{S14}$$

$$\xi(\boldsymbol{n}) = \left(\frac{\cos^2\theta \sin^2\varphi}{\beta_1^2} + \frac{\sin^2\theta \sin^2\varphi}{\beta_2^2} + \frac{\cos^2\varphi}{\beta_3^2}\right)^{-\frac{1}{2}}, \tag{S15}$$

where ξ_i and β_i $(i = 1,2,3)$ stand for the initial moduli and fiber nonlinearity (power coefficient) along three principal axes of the ellipsoidal fibers. In the superior and inferior CEPs, the fibers primarily exhibit orientation within planes parallel to the vertebral bodies and the NP. Along the lateral and anterior-posterior directions, these fibers share the same elastic modulus $(\xi_1 = \xi_2)$. However, in the axial direction of the IVDs, a significantly smaller elastic modulus was assumed, with $\xi_3 = 0.1\xi_1$. The fiber nonlinearity was considered to be direction-independent, represented as $\beta(\boldsymbol{n}) = \beta_c$, where β_c is a constant. Through a regression analysis by fitting against experimental data due a tensile test, we determined the values of $\xi_1 = \xi_2 = 10\,\xi_3 = 7.01$ MPa and the fiber nonlinearity constant $\beta_c = 2.88$ (DeLucca et al., 2016).

S5 The scaling factor and concentration factor

Table S1: The scaling factor s_f and the concentration factor b for each elastic fiber's family. Parameter values for the 45° and 135° fiber families were averaged. The spatial distributions of the scaling factor were obtained by linear interpolated along radial direction for both lamella and inter-lamellae layers. Abbreviations: AO (Anterior outer); AI (Anterior inner); PO (Posterolateral outer); PI (Posterior inner); LM (Lamella); ILM (Inter-lamellae).

Principal direction	0°	45°	90°	135°
The scaling factor (s_f^{II})				
AO-LM	8.33	1.00	0.21	1.00

AO-ILM	0.00	1.24	0.76	1.24
AI-LM	13.13	1.00	0.21	1.00
AI-ILM	0.00	0.00	11.60	0.00
PO-LM	8.33	1.00	0.21	1.00
PO-ILM	0.00	1.24	0.76	1.24
PI-LM	9.82	1.00	0.21	1.00
PI_ILM	0.00	0.00	7.42	0.00
The concentration factor (*b*)				
AO-LM	62.2	23.2	32.0	35.2
AO-ILM	79.5	218.5	133.2	80.5
AI-ILM	19.8	0.0	14.6	0.0
PO-LM	62.2	23.2	32.0	35.2
PO-ILM	79.5	218.5	133.2	80.5
PI-ILM	29.5	0.0	14.3	0.0
